# Supplementary material for: Estimating the effect of social inequalities on the mitigation of COVID-19 across communities in Santiago de Chile
Source: Nat Commun. 2021 Apr 23;12:2429. doi: 10.1038/s41467-021-22601-6 (PMC8065143; doi:10.1038/s41467-021-22601-6)
Supplement: Supplementary file 2 — Supplementary Information [file 41467_2021_22601_MOESM2_ESM.pdf]

Supplementary information for *Estimating the effect of social inequalities on the mitigation of COVID-19 across communities in Santiago de Chile*

April 5, 2021

Contents

1 Sensitivity analysis to data and modeling parameters 2

2 Sensitivity to the modeling framework 6

3 Homogeneous NPIs Effect - Counterfactual Scenario 12

4 Metapopulation Model with Age Structure 14

5 Mobile Phone Users Geographical Distribution 17

1 Sensitivity analysis to data and modeling parameters

In this section we provide a sensitivity analysis on the data used for model calibration, on model parameters, on the correlation between mobility changes and sociodemographics, and finally on the number of days needed to reach N infections according to our estimates and official surveillance in different comunas.

Deaths Data for Calibration. We calibrate the model using COVID-19 deaths data issued by the Department of Statistics of the Chilean Minister of Health (DEIS) [1]. In fact, while there are biases in any indicator, the number of confirmed cases is arguably one of the most affected by varying reporting rates. Testing capabilities and testing strategies that target only severe symptomatic individuals induce high levels of underreporting which are also time dependent. Though not perfect, deaths (and hospitalizations) are less prone to under-reporting than infections. The DEIS dataset includes both "confirmed" (i.e. supported by a clinical test, ICD code: U07.1) and "suspected" deaths (i.e. supported only by symptoms, ICD code: U07.2). In Supplementary Figure 1A we compare 2020 all-cause deaths with historical median and 95% confidence intervals. We observe that, excluding only COVID-19 confirmed from 2020 deaths we still obtain a significant anomaly. Excluding also suspected, instead, we obtain a trend that is within confidence intervals. For this reason, we decide to calibrate the model considering both COVID-19 confirmed and suspected deaths.

Here, we perform also calibration considering only confirmed COVID-19 deaths. In Supplementary Figure 1B we observe that the estimates for R_0 obtained in this case do not change significantly (median R_0 of 2.58, 95% CI: $[2.50, 2.64]$). Moreover, this approach leads to a worse fit of the data (median absolute percentage error of 28% with respect to the 12% obtained considering also suspected deaths).

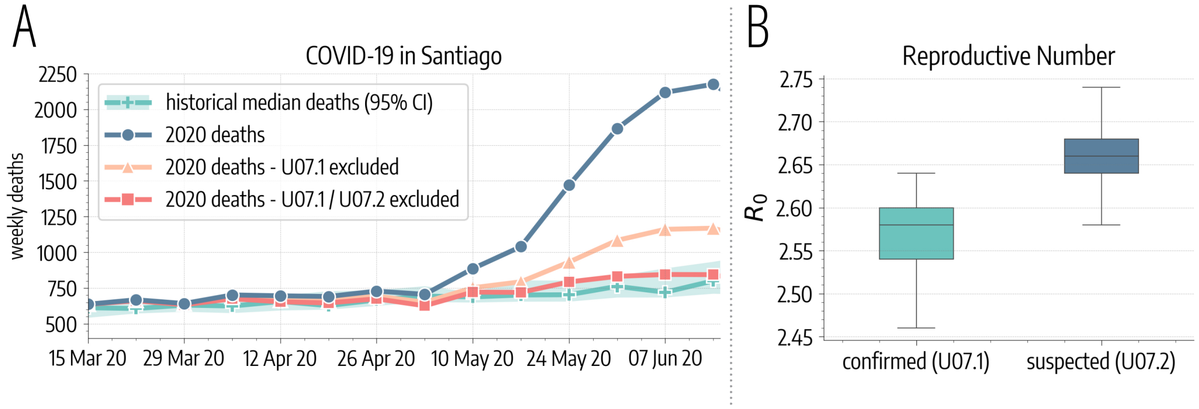

Supplementary Figure 1: **Impact of COVID-19 on mortality rate in Santiago.** A) We plot 2020 all-cause weekly deaths in the comunas of Santiago and the historical median with 95% confidence interval computed on 2010-2019 data. We report also 2020 deaths time series excluding only COVID-19 confirmed (ICD code: U07.1) and also suspected (ICD code: U07.2) deaths. B) R_0 estimates considering only confirmed and also suspected COVID-19 deaths (based on $n = 1,000$ samples from the posterior distribution). In the boxplots, the centre of the boxes indicates the median, the bounds indicate the interquartile range (IQR) (i.e., the range between first quartile, Q1, and third quartile, Q3), and the whiskers indicate the minimum and the maximum defined respectively as $Q1 - 1.5IQR$ and $Q3 + 1.5IQR$.

Incubation and Infectious Period. In the main text we considered an incubation period (T_{IC}) of 4 days and an infectious period (T_{IF}) of 2.5 days. Here, we run simulations where we vary these parameters and we observe the impact on our findings. In particular, we consider estimated R_0, prevalence and increase in deaths without the full lockdown for longer incubation ($T_{IC} \in [5, 6]$ days) and infectious periods ($T_{IF} \in [3, 3.5]$ days). In Supplementary Figure 2 we represent results of the sensitivity analysis. From Supplementary Figure 2A we observe that the median R_0 for the different parameters explored remains in the range $[2, 3]$. Also, from Supplementary Figure 2B we note that the median projected prevalence as of August 1, 2020 in Santiago varies of at most 5.9%. Finally, the results of the counterfactual analysis in which we do not introduce the full lockdown are very robust across different parameters explored. Indeed, the median projected increase in deaths as of August 1, 2020 varies of at most 3.0%.

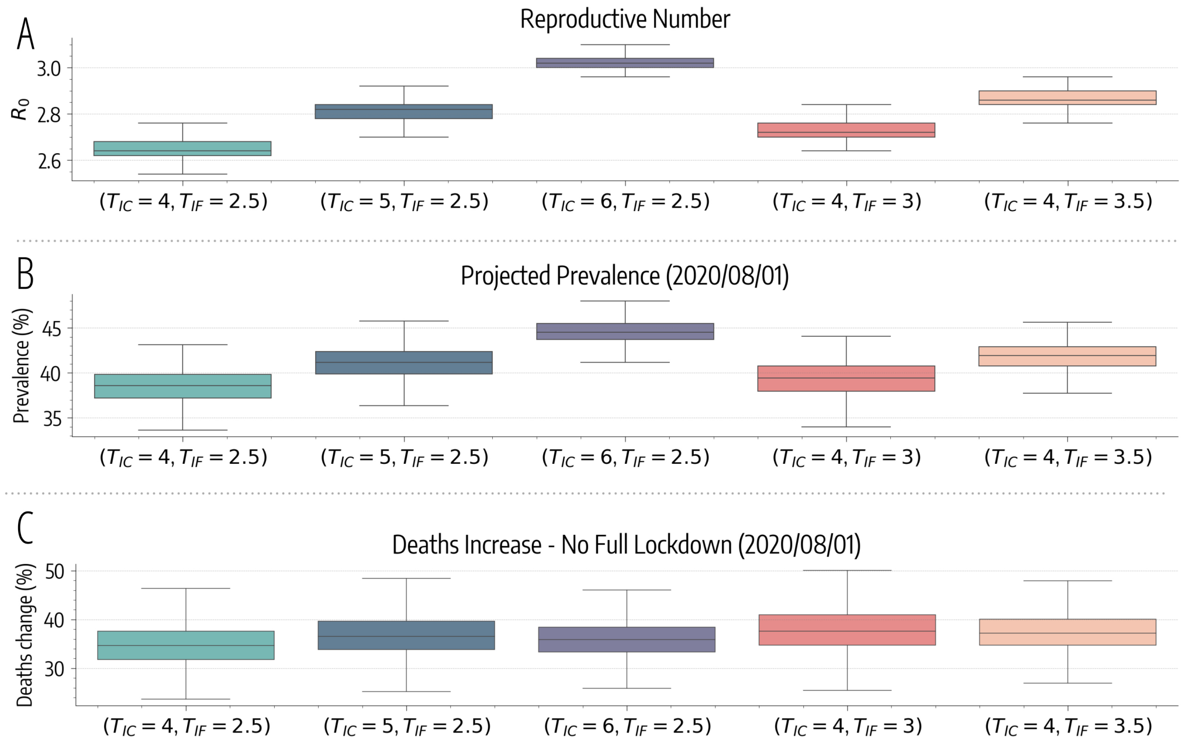

Supplementary Figure 2: **Results of sensitivity analysis** A) Basic reproductive number estimates for different parameters explored in the sensitivity analysis (based on $n = 1,000$ samples from the posterior distribution). B) Model projections of prevalence in Santiago as of August 1, 2020 for different parameters (based on $n = 5,000$ stochastic realizations). C) Increase in deaths (in percentage) as of August 1, 2020 without the full lockdown for different parameters (based on $n = 5,000$ stochastic realizations). In the boxplots, the centre of the boxes indicates the median, the bounds indicate the interquartile range (IQR) (i.e., the range between first quartile, Q1, and third quartile, Q3), and the whiskers indicate the minimum and the maximum defined respectively as $Q1 - 1.5IQR$ and $Q3 + 1.5IQR$.

ABC Tolerance. In the main text we used the median absolute percentage error as an error metric for the ABC calibration with a tolerance of $\epsilon = 20\%$. We here repeat the calibration with two different ϵ (15%, 25%) as a sensitivity check. Results are displayed in Supplementary Figure 3. We observe that changes in the tolerance do not impact significantly the posterior distribution of the basic reproduction number R_0.

Mobility and Sociodemographics. In the main text, we showed that the change in mobility correlates with the Human Development Index (HDI) of different comunas. Here, we show that this pattern holds for other economic and development indicators too. In particular, we consider separately the components of the HDI, namely the Life Expectancy Index (LEI), the Education Index (EI), and the Income Index (II) [2]. In Supplementary Figure 4 we see that the correlation between these indicators and the average mobility drop after 16/03 of the 37 comunas considered is still high, significant, and consistent with the results presented in the main text for the HDI.

Also, in the main text we showed the significant correlation between HDI and the average percentage decrease in mobility after March 16th, 2020. Here, we show that this finding hold also for absolute mobility decreases. This sensitivity check is needed since a higher percentage decrease does not imply necessarily a lower mobility after the introduction of NPIs (for example, the mobility baseline of wealthier comunas can be generally higher). We define the outflow as the average number of outgoing travels per device observed for a comuna during a given period. In Supplementary Figure 5 we represent (left) the outflow before March 16th, 2020 versus the HDI of different comunas. We observe no significant correlation ($\rho = 0.01$, $p = 0.94$). On the right, instead we represent the scatter plot of the absolute

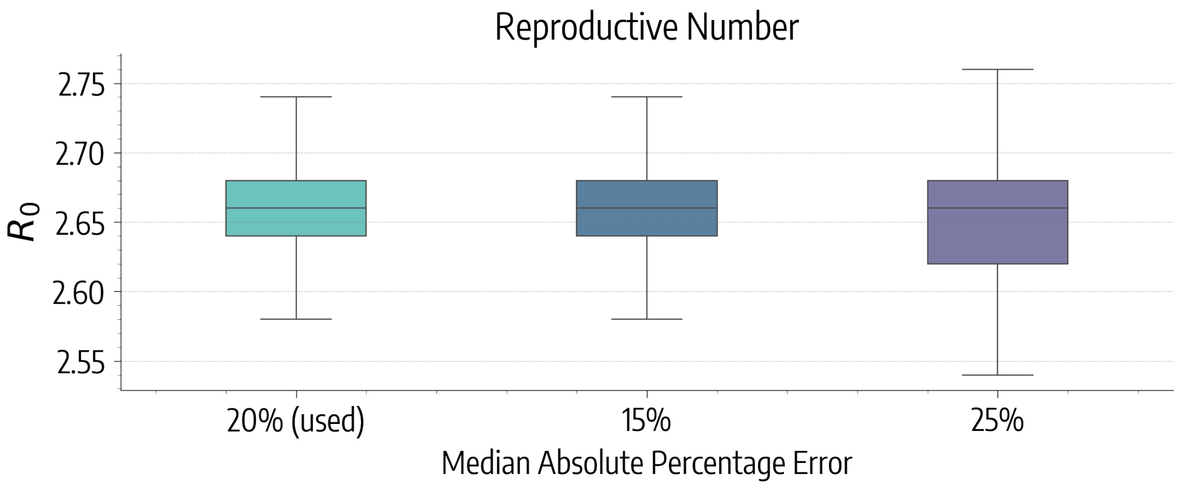

Supplementary Figure 3: **Different ABC tolerance.** We show the calibrated R_0 for different ABC tolerance: 20% (used in the main text), 15%, and 25% (based on $n = 1,000$ samples from the posterior distribution). In the boxplots, the centre of the boxes indicates the median, the bounds indicate the interquartile range (IQR) (i.e., the range between first quartile, Q1, and third quartile, Q3), and the whiskers indicate the minimum and the maximum defined respectively as $Q1-1.5IQR$ and $Q3+1.5IQR$.

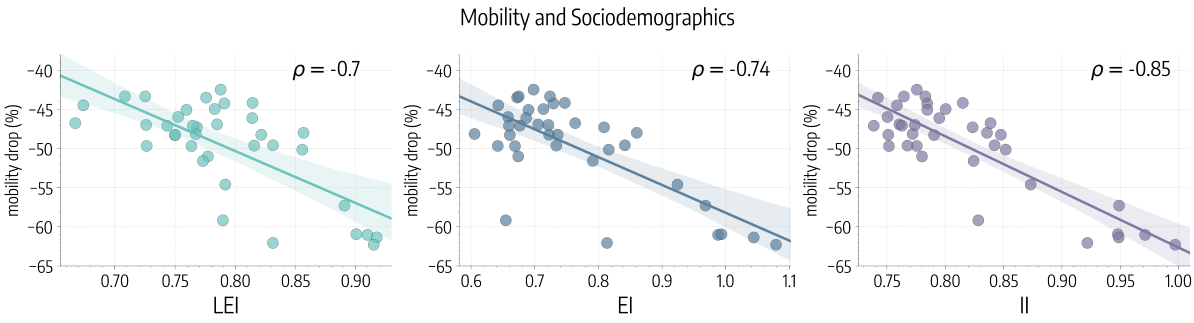

Supplementary Figure 4: **Correlation between mobility and sociodemographics.** We represent the scatter plot and the correlations between average percentage drop in mobility after 16/03 and the Life Expectancy Index (LEI), the Education Index (EI), and the Income Index (II). We display regression line, 95% CI and the Pearson correlation coefficient ρ.

reduction in outflow before/after March 16th, 2020 versus the HDI of comunas. In this case we find a strong, positive, and significant correlation ($\rho = 0.55$, $p < 0.0001$), hinting that wealthier comunas reduced more their mobility also in absolute terms.

Projected cases. In the main text, we assessed the performance of the model comparing via the Kendall rank correlation coefficient the number of days (since 2020/01/01) needed to reach 200 infections in different comunas according to our projections and official surveillance. We found a good, significant correlation and we remarked that dates estimated through modeling were generally earlier to those officially reported. In Supplementary Figure 6 is shown that this pattern holds also for different numbers of infections. In particular, we consider the amount of days needed to reach 50, 100, 200, and 500 infections.

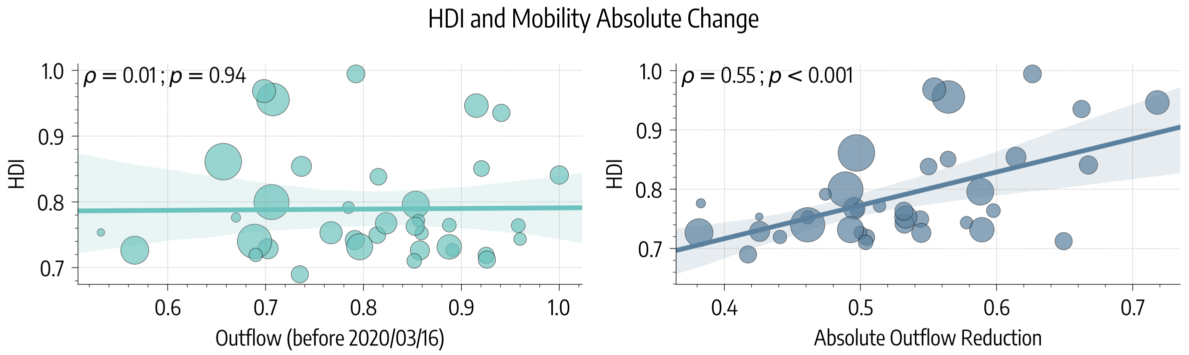

Supplementary Figure 5: **HDI and absolute mobility changes.** On the left, scatter plot of outflow (normalized) before March 16th, 2020 versus HDI of different comunas. On the right, scatter plot of the absolute reduction in outflow after March 16th, 2020, versus HDI of different comunas. Points size is scaled according to population. We display regression line, 95% CI, the Pearson correlation coefficient ρ and p-values.

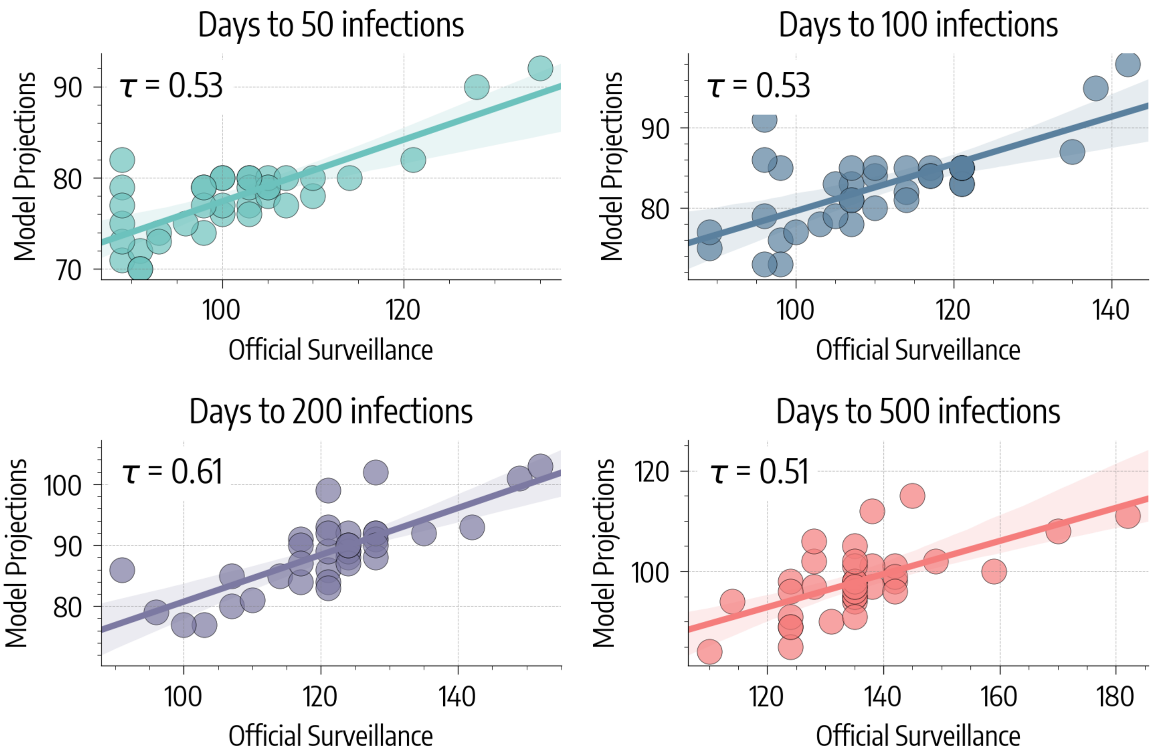

Supplementary Figure 6: **Days to reach N infections in different comunas.** We consider the number of days since 2020/01/01 needed to reach 50, 100, 200, and 500 infections in different comunas as projected by our model and as confirmed by official surveillance. We display regression line, 95% CI and the Kendall rank correlation coefficient τ.

Supplementary Table 1: **Alternative compartmental structure - Parameters**

Parameter	Value	Source
Latent period, ϵ (time spent in E)	3.7 days	[8, 9]
Prodromal stage, δ (time spent in P)	1.5 days	[8, 9]
Fraction of asymptomatic carriers, r	0.2, 0.4	[10, 11]
Ratio of transmission rate of I vs P/A, α	0.55	[12]
Infectious period, μ (time spent in A, I)	2.5 days	[13, 14]

2 Sensitivity to the modeling framework

Alternative Compartmental Structure. In the main text, we used a SLIR compartmentalization setup. Although this approach has been previously used in several modeling studies focused on COVID-19 [3–5], we acknowledged the simplicity of the compartmental structure as one of the possible limitations. Indeed, the dynamics of infectious diseases is much more complex than the one depicted by a $SLIR$ model. In the context of COVID-19, both pre-symptomatic and asymptomatic transmissions have become of great concern. To include these aspects in our analysis, we propose the following alternative compartmental structure and we repeat the analyses of the main text. The findings are confirmed also considering different compartmental structures.

Susceptibles (S) individuals after interacting with infectious transit to the Latent compartment (L). After the latent period (ϵ^{-1}), L individuals enter the prodromal phase (P) that refers to pre-symptomatic and infectious individuals. These then evolve either in the asymptomatic (A) or the symptomatic stage (I) at rate δ (the length of time including L and P stages is the incubation period). The fraction of individuals transiting towards I or A is regulated by the probability of being asymptomatic r. Both I and A individuals after the infectious period enter the Recovered compartment (R_I, R_A). We compute deaths considering only on the Recovered resulting from the I compartment (i.e., symptomatic). The infectious compartments are P, A, I. We assume that P and I have lower infectiousness with respect to symptomatic I. We indicate the ratio of the transmission rate of I and P, A infectious with $\alpha < 1$. In the following, we will refer to this model as $SLPIAR$ and to the model used in the main text simply as $SLIR$. It is also important to notice that the $SLPIAR$ model has a new definition of the basic reproduction number $R_0 = \beta \cdot \rho(C) \cdot (\frac{\alpha}{\delta} + \frac{1-\alpha}{\mu} + \frac{\alpha \cdot r}{\mu})$. In Supplementary Figure 7 we report a schematic representation of the $SLPIAR$ compartmental structure. Similar approaches have been employed in other modeling studies [6, 7]. In Tab. 1 we report the values of the parameters used and the related sources. In particular we explore two values of the probability of being asymptomatic $r = 0.2, 0.4$.

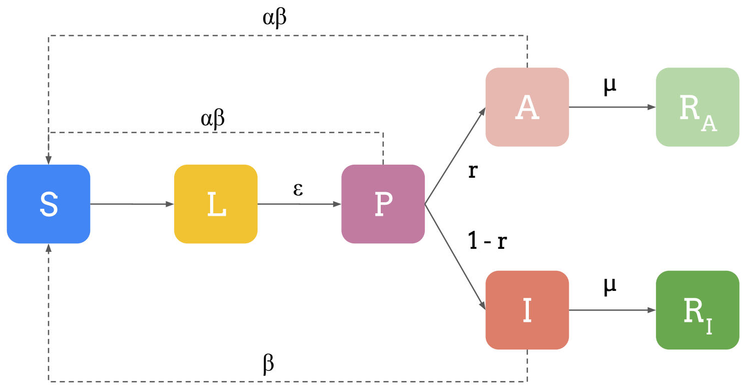

Supplementary Figure 7: **Alternative compartmental structure.** With respect to the structure used in the main text, we added the compartments P and A to include both pre-symptomatic and asymptomatic transmission.

We repeat part of the analyses presented in the main text with this new compartmental setup. We perform also for the $SLPIAR$ model an Approximate Bayesian Calibration on weekly deaths using the median absolute percentage error as an error metric. For convergence reason, we used a threshold of 30% with respect to 20% used in the main text. Results are summarized in Supplementary Figure 8. In panel

A we represent a boxplot for the fitted values of R_0. In the case of the $SLIR$ model we obtain a median R_0 of 2.66 (95% CI: $[2.58, 2.72]$), while for the $SLPIAR$ model we obtain a median of 2.92 (95% CI: $[2.86, 3.00]$) and 3.02 (95% CI: $[2.98, 3.10]$) for $r = 0.2$ and $r = 0.4$ respectively. These differences should not surprise. Indeed, the basic reproductive number is strongly dependent on the modeling setup and small variation are expected across different approaches. In panel B, we represent the reported weekly deaths along with simulated weekly deaths from the three models. Across the board, also the $SLPIAR$ model is able to reproduce well the evolution of deaths in the region. In terms of median absolute percentage error, we obtain an average of 23% for $r = 0.2$ and of 28% for $r = 0.4$. In the main text, for a SLIR model, we obtained 12%. In panel C, we represent the correlation between reported and simulated prevalence in different comunas at the 1st of August, 2020. Similarly to what obtained in the main text ($SLIR$ model), we obtain also for the $SPLIAR$ model a high significant Pearson correlation coefficient of 0.86 when $r = 0.2$ and of 0.87 when $r = 0.4$. Finally, in panel D we represent the simulated attack rate and the HDI of different comunas. Also for the $SPLIAR$ model we obtain a negative significant correlation of -0.69 for both values of r.

Model without mobility coupling. In the main text we presented our metapopulation approach to model the spreading of SARS-CoV-2 in Santiago de Chile. In this setting, the comunas are represented as distinct subpopulations connected through the movements of people in a metapopulation network. We showed that the force of infection (i.e., the probability of infection) in different comunas is a function of the mobility coupling between them (more details in the following Section 4). To assess the importance of accounting for such coupling, we consider here, as comparison, a simpler modeling approach in which we completely disregard the mobility between comunas. Said differently, we study the different comunas as 37 disconnected populations. As a result, the force of infection reduces to a much simpler expression. Since we also consider age-structured populations, for age group k in comuna j it reads as:

$$\lambda_j^k = \beta \sum_{k'} C^j_{kk'} \frac{I^j_{k'}}{N^j_{k'}} \tag{1}$$

Despite we completely drop the mobility network, to account - at least partially - for the effects of restrictions, we still take into account the contacts reduction parameters $r_j^{partial}$ and r_j^{full} derived in the main text and that multiply the contacts matrix of comuna j on the establishment, respectively, of the partial and full lockdown. With this setup, we repeat the calibration step on total weekly deaths observed in Santiago over the period March-August 2020 through the ABC procedure.

We obtain a median R_0 of 2.72 (95% CI: $[2.60, 2.84]$), compatible with the result obtained in the main text. However, from Supplementary Figure 9 immediately emerges that the obtained model's projections interpolate the reported data with an inferior performance with respect to the results from the metapopulation approach. More quantitatively, we obtain a median absolute percentage error of 38% with respect to 12% obtained for the metapopulation model. Furthermore, we obtain a drop in the correlation between simulated and reported prevalence in different comunas (from ρ=0.84 to ρ=0.63). These findings highlight the importance of consider the coupling between areas via mobility to capture the spatial spread of SARS-CoV-2 in the Metropolitan Region of Santiago.

Single Population Model. Here, we study a simpler approach modeling Santiago de Chile as a single population rather than a network of connected subpopulations (i.e., comunas). This methodology has been widely proposed in the literature to study the COVID-19 pandemic at different geographical resolutions such as cities [15], regions [6], or countries [16]. In the same spirit of what presented above, the goal is to evaluate the performance of simpler approaches and alternative data sources. We consider the population of Santiago divided into the 16 age groups and we consider the country-specific contacts matrix to describe the rates of contacts between age brackets. The disease dynamics is modeled through the SLIR framework used in the main text. In this settings, the force of infection for age group i at time t reduces to:

$$\lambda_i(t) = \beta \sum_j \frac{C_{ij}(t)}{N_j} I_j(t). \tag{2}$$

More in detail, we study two distinct models. In the first one, we characterize contacts reduction in Santiago, following NPIs, using our mobile phone data. In the main text, we computed contacts reduction parameters for each comuna j for the partial and the full lockdown: $r_j^{partial}$, and r_j^{full}. Here, we compute the weighted average (according to the population of different comunas) of these parameters to obtain two, overall, contacts reduction parameters: $r^{partial}$, and r^{full}. Following the approach of the

main text, between 16/03 and 15/05 (partial lockdown) we multiply the contacts matrix by $r^{partial}$, and by r^{full} after 15/05 (full lockdown).
In the second model, we use alternative data sources to evaluate contacts reductions in different locations following the restrictions. Indeed, the contacts matrix is made up of four contributions: contacts that happen at school, workplace, home, and other locations. In general, the overall contacts matrix $C(t)$ is a linear combination of the four terms:

$$C(t) = \tilde{\omega}_h(t) home + \tilde{\omega}_s(t) school + \tilde{\omega}_w(t) work + \tilde{\omega}_o(t) otherlocations \tag{3}$$

Where the $\tilde{\omega}_l(t)$ are the location-specific, time-varying contacts reduction coefficients. We use the Google COVID-19 Community Mobility Report [17] to characterize contacts variations at home, workplace, and other locations. Let $\omega_l(t)$ be the general entry of the Report, representing the percentage change of the number of visits (with respect to a pre-pandemic baseline) to specific location l on day t. We turn this quantity into a contacts reduction parameter as follows: $\tilde{\omega}_l(t) = (1 + \omega_l(t)/100)^2$. Indeed, the number of contacts between individuals scales with the square of the number of individuals. For contacts reduction in workplaces we consider the field "workplaces percent change from baseline" of the Report, and the average of the fields "retail and recreation percent change from baseline" and "transit stations percent change from baseline" for other locations. For contacts at home we consider the field "residential percent change from baseline" but we omit in this case the square in the previous formula since this entry is a proxy for additional time spent at home rather than actual visits. Finally, we model contacts reduction at school using the Oxford COVID-19 Government Response Tracker [18, 19]. We consider the quantity "C1 School closing" from this dataset, an ordinal index that goes from a maximum of 3 (i.e., requires school closing at all levels), to a minimum of 0 (i.e., no restrictive measures in place). We turn this quantity into a contacts reduction parameter for school as follows: $\tilde{\omega}_s(t) = 1 - \texttt{C1 School closing}/3$.
In both models we explore the same free parameters mentioned above: the basic reproductive number R_0 and the delay in deaths Δ. The calibration is performed also in this case through Approximate Bayesian Computation rejection algorithm.

In Figure 10 we show weekly deaths as reported by official surveillance along with the simulated time series by the two models in Santiago (median and 95% CI). We observe that the model with contacts estimated on our mobile phone data performs significantly better with respect to the one using other data sources (median absolute percentage error of 18% with respect to 43%). Nonetheless, it has a lower performance than the metapopulation model presented in the main text, which shows a median absolute percentage error of 12%. It is worth stressing that these simpler models are agnostic to the differences in disease burden between comunas. Understanding such heterogeneities/differences, which are also clear in the epidemiological data, is a key aspect of our study.

Comparing models' performance. In Supplementary Table 2 we summarize the performance of all models considered by comparing their median absolute percentage errors. The best model is the one presented in main text: a SLIR metapopulation framework informed by the mobile phone data to estimate variations in mobility and contacts. The second is the model that considers the whole metropolitan area as a single, age-structured, population and uses mobile phone data to estimate variations in the contact matrix. Despite the good performance, by construction, this model is agnostic about the heterogeneities in the spreading patterns of the virus across comunas. The third is the metapopulation model based on a SLPIAR compartmentalization for $r = 0.2$. This is followed closely by the same modeling structure but for $r = 0.4$. In fifth position we find the model that considers comunas as separated, age-structured, subpopulations hence neglecting the mobility coupling between them. In last position we find the model that studies the whole metropopolitan area as single, age-structured, population and adopts Google Mobility Reports as well as the Oxford COVID-19 Government Response Tracker to estimate the variation in the contact matrices.

	**MdAPE**
Metapopulation Models with Mobility	
**SLIR**	***12%***
**SLPIAR (r=0.2)**	*23%*
**SLPIAR (r=0.4)**	*28%*
Comunas as separated subpopulations	
**No Mobility**	*38%*
Single Population Models	
**Single Pop. (TEF)**	*18%*
**Single Pop. (Alternative data)**	*43%*

Supplementary Table 2: **Performance of different models**. We report here the performance (in terms of median absolute percentage error) of the different models considered

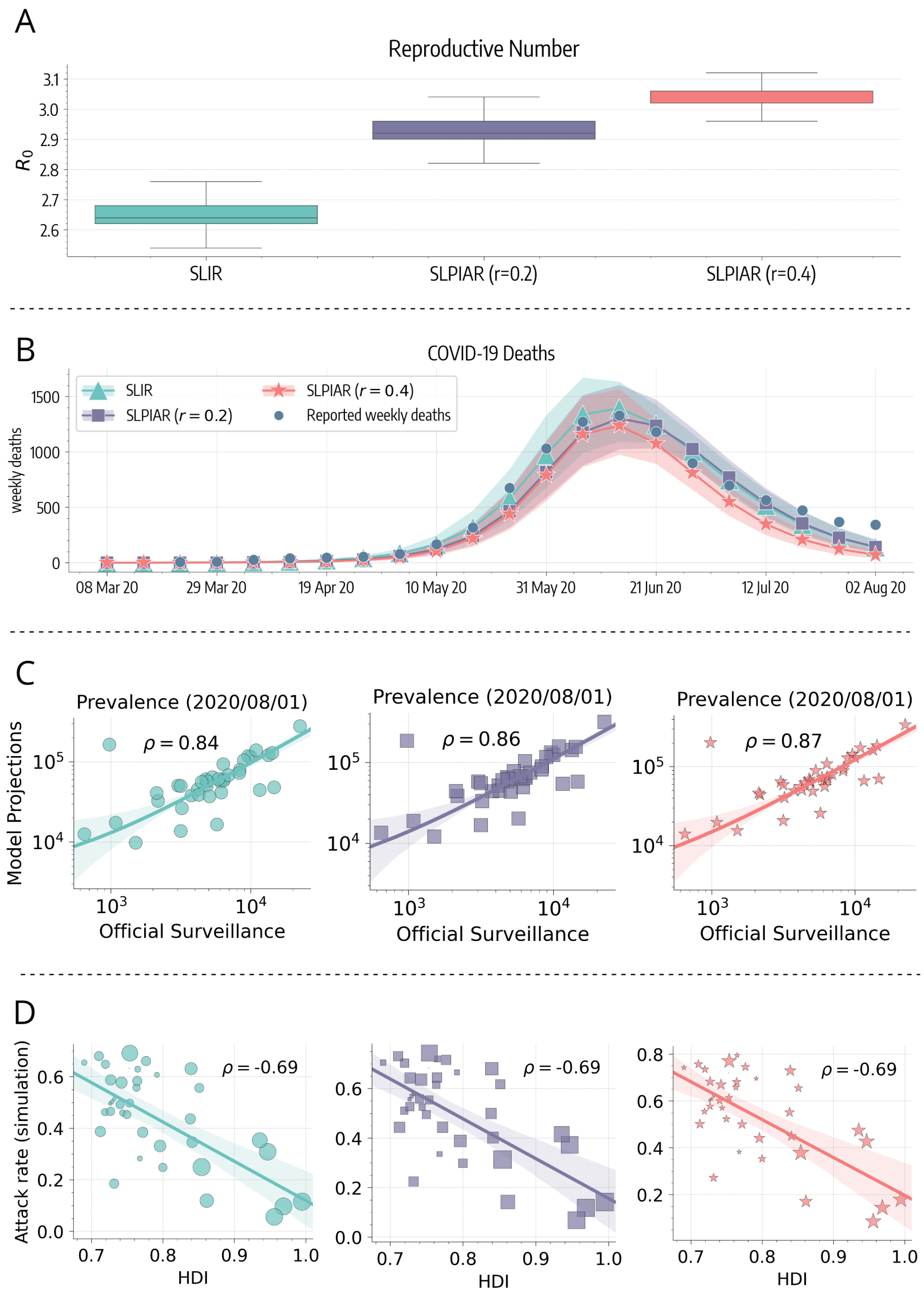

Supplementary Figure 8: **Alternative compartmental structure - Sensitivity analysis.** We compare the results obtained for the alternative compartmental structure (for two values of r) with those obtained in the main text. A) Fitted R_0 (based on $n = 1,000$ samples from the posterior distribution). In the boxplots, the centre of the boxes indicates the median, the bounds indicate the interquartile range (IQR) (i.e. the range between first quartile, Q1, and third quartile, Q3), and the whiskers indicate the minimum and the maximum defined respectively as $Q1 - 1.5IQR$ and $Q3 + 1.5IQR$. B) Fitted and reported weekly deaths (median and 95% CI). C) Real and projected prevalence on 2020/08/01 (regression line and 95% CI displayed; the Pearson correlation coefficient ρ is reported in legend). D) Correlation between attack rate and HDI in different comunas (regression line and 95% CI displayed; the Pearson correlation coefficient ρ is reported in legend).

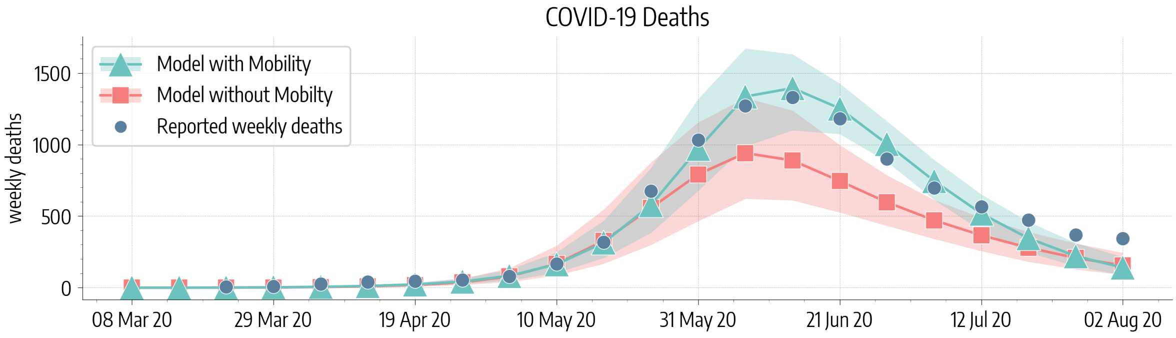

Supplementary Figure 9: **Model without mobility - Deaths fit.** We compare the median and 95% CI of weekly deaths projected by the simpler model with those projected by the metapopulation model with mobility and with real observed data.

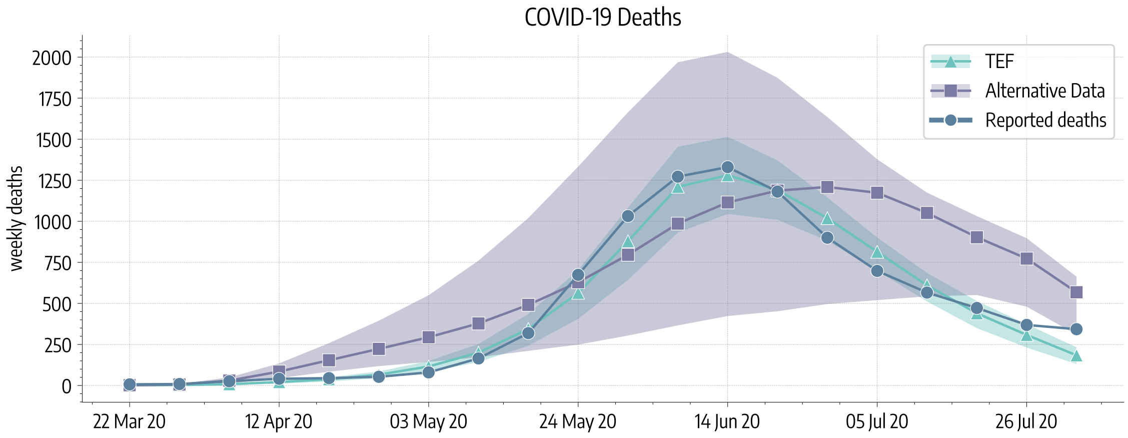

Supplementary Figure 10: **Single population models**. We consider a simpler approach in which we model Santiago as a single population. In the first model (TEF) we use our mobility dataset to infer contacts changes in Santiago following restrictions. In the second model (Alternative data) we insights from the Google COVID-19 Community Mobility Report and the Oxford Policy Tracker. We report median and 95% CI for the weekly deaths projected by the two models along with weekly deaths reported by official surveillance.

3 Homogeneous NPIs Effect - Counterfactual Scenario

In the main text we present a hypothetical scenario in which we assign to all comunas the average decrease in mobility rates and contacts observed after the partial lockdown for the 4th quartile of HDI (i.e. 25% comunas with higher HDI). In Supplementary Figure 11A we represent the percentage change in total COVID-19 deaths and cases as of 2020/05/15, date of the 2nd lockdown. We observe that the effect on the spreading is significant: -83.8% (95% CI: [-77.6%, -88.6%]) fewer cases and -70.5% (95% CI: [-55.0%, -80.9%]) fewer deaths. In Supplementary Figure 11B we represent the percentage change in COVID-19 cases as of May 15th in different comunas. We observe that, in general, the uniform reduction in mobility and contacts brings benefits also to comunas with higher HDI. This hints that a more equal distribution of behavioral responses to the NPIs in place would be advantageous not only for the most vulnerable but to the community as a whole.

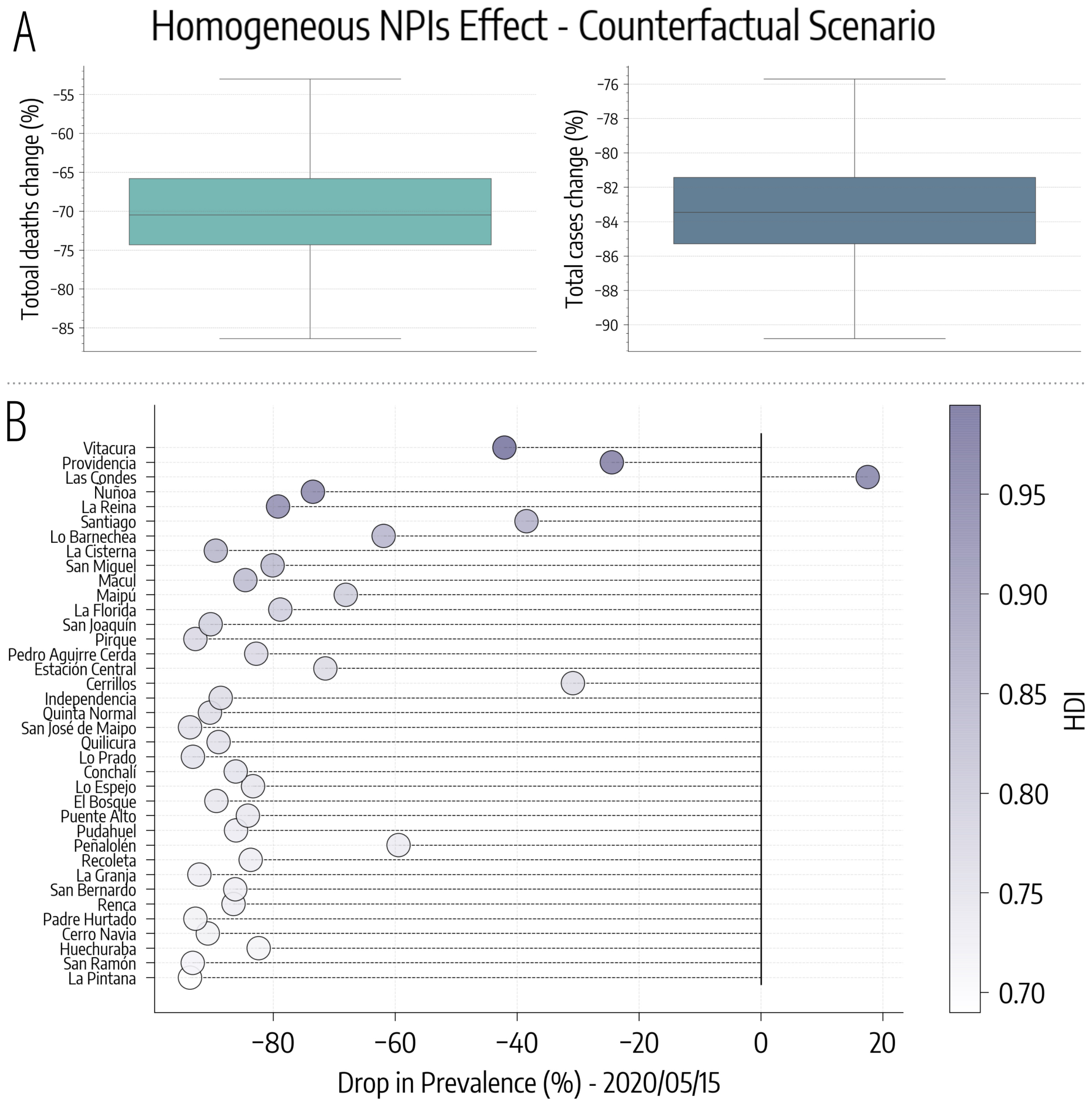

Supplementary Figure 11: **Homogeneous NPIs Effect - Counterfactual Scenario.** A) Drop (%) in total number of COVID-19 deaths and cases as of 2020/05/15, date of the 2nd lockdown (based on $n = 5,000$ stochastic realizations). In the boxplots, the centre of the boxes indicates the median, the bounds indicate the interquartile range (IQR) (i.e., the range between first quartile, Q1, and third quartile, Q3), and the whiskers indicate the minimum and the maximum defined respectively as $Q1 - 1.5IQR$ and $Q3 + 1.5IQR$. b) Drop (%) in COVID-19 cases in different comunas (based on $n = 5,000$ stochastic realizations).

4 Metapopulation Model with Age Structure

The model presented here is largely based on Ref. [20]. We consider N comunas and K age groups. We introduce the matrix $C^j \in \mathbb{R}^{K\times K}$ whose element $C^j_{kk'}$ indicates the contacts rate between age group k and k' in comuna j. Similarly, we introduce the matrix $\boldsymbol{\sigma} \in \mathbb{R}^{N\times N}$, whose element σ_{ji} indicates the mobility rate from comuna j to i. We also define τ as the return rate of travelling individuals to the home comuna. We consider a $SLIR$ compartmentalization setup with traveling Infectious, analogous to the case described in the main text (Tab. 3). In the next paragraphs we present the integration of mobility dynamics, the derivation of the force of infection, and how mobility restrictions are reflected in our model.

Time scale separation and integration of mobility. Simulations and disease progression evolve with the time scale of the day. However, the mobility between comunas happens at a faster pace (set by the return rate τ). This is clear in Supplementary Figure 12 where we plot the probability density function of trips' duration. To simplify the visualization we do not show the density for trips lasting more than 7 days (about 0.2% of the travels). We can see that around 85% of inter-comunas travels happen within 8 hours. Furthermore, the average trip duration of 4.5 hours and the probability of observing travels lasting more than 24 hours is smaller than 3% (note the logarithmic scale on the y-axis).

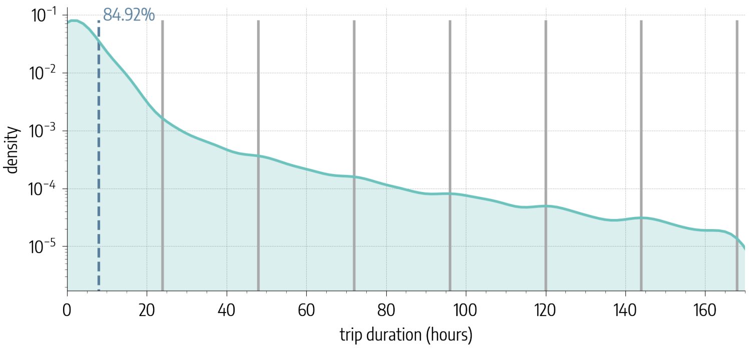

Supplementary Figure 12: **Travel duration.** We represent the probability density function of trip duration (expressed in hours). Vertical dashed line indicate 8 hours, while solid vertical lines indicate days progression. The probability density function is computed from about $97M$ travels between March and June 2020 in Santiago Metropolitan Region.

Considering this observation we use a time-scale separation technique that allows to integrate the short-time mobility dynamics and factor in its effective contributions to the slower processes (in our case, the progression of the disease) [21]. We note that this time-scale separation is exact only in the limit of infinitely fast mobility dynamics ($\tau^{-1} \to 0$). However, it remains valid as long as the mobility time scale is much smaller than the typical transition rates of the disease dynamics. Here, we aim to consider the temporal evolution of number of individuals in different comunas, connected by mobility flows, and to evaluate their equilibrium configuration with respect to the slower dynamics (i.e, the progression of the simulation and of the disease).

For a general compartment X (here $X \in [S, L, I, R, N]$), we indicate with $X^k_{jj}(t)$ the number of people from comuna j (in age group k) actually located in j at time t, and with $X^k_{ji}(t)$ the number of people from j (in age group k) located in a connected comuna i at time time t [22]. For consistency, hold the following relation:

$$X^k_j = X^k_{jj}(t) + \sum_i X^k_{ji}(t) \tag{4}$$

The number of people living in comuna j, in compartment X, and age group k (X^k_j) changes at a time-scale driven by the transition rates of the disease and by the temporal resolution of the simulations (i.e., a day). Hence, it is considered at equilibrium respect to the faster dynamics driving the mobility flows. In fact, the time t in the equation captures the time-scale of mobility which takes places *within* a day. For $X^k_{jj}(t)$ and $X^k_{ji}(t)$ we can write the following rate equations:

$$d_t X^k_{jj}(t) = -\sum_i \sigma_{ji} X^k_{jj}(t) + \tau \sum_i X^k_{ji}(t) \tag{5}$$

$$d_t X^k_{ji}(t) = \sigma_{ji} X^k_{jj}(t) - \tau X^k_{ji}(t) \tag{6}$$

From Eq. 4 we derive $\sum_i X^k_{ji}(t) = X^k_j - X^k_{jj}(t)$. We substitute this expression in Eq. 5 and we define the total mobility rate of comuna j as $\sigma_j = \sum_{i \neq j} \sigma_{ji}$:

$$d_t X^k_{jj} = \tau X^k_j - (\sigma_j + \tau) X^k_{jj}(t) \tag{7}$$

We solve Eq. 7 for $X^k_{jj}(t)$:

$$X^k_{jj}(t) = \frac{\tau X^k_j}{\sigma_j + \tau} + \left[X^k_{jj}(0) - \frac{\tau X^k_j}{\sigma_j + \tau} \right] e^{-\tau(1+\sigma_j/\tau)t} \tag{8}$$

The relaxation to the equilibrium value of X^k_{jj} is controlled by the exponential term via the characteristic time $[\tau(1 + \sigma_j/\tau)]^{-1}$. This expression reduces to $1/\tau$ in case $\sigma_j/\tau << 1$. The total mobility rate σ_j is always smaller than 1 - since not everyone is travelling - and it is generally much smaller than $\tau = 3 \ day^{-1}$ (that we set to account for commuting as well as other movements). We can then approximate the characteristic time to $1/\tau$. This is in turn smaller than the time-scale of simulation and disease progression (in the case of COVID-19, both the latency ϵ^{-1} and infectious μ^{-1} period are larger than 1 day). Therefore, when t tend to the time-scale of the simulation we can consider $X^k_{jj}(t)$ relaxed to its equilibrium value:

$$X^k_{jj} = \frac{X^k_j}{1 + \sigma_j/\tau} \tag{9}$$

Similarly, we derive the equilibrium value for $X^k_{ji}(t)$. We take Eq. 6 and we impose the equilibrium condition $\sigma_{ji} X^k_{jj} - \tau X^k_{ji} = 0$. Then, substituting the result just derived, we obtain the equilibrium value also for X^k_{ji}:

$$X^k_{ji} = \frac{X^k_j \sigma_{ji}/\tau}{1 + \sigma_j/\tau} \tag{10}$$

Force of infection. We define the force of infection λ^k_j as the probability that a susceptible individual in age group k and in comuna j transit to the Latent compartment in the unit of time. This force of infection has two contributions, from the contacts occurred in the home comuna (λ^k_{jj}) and in other connected comunas (λ^k_{ji}). These can be written down as:

$$\lambda^k_{jj} = \frac{\beta}{N^*_j} \left[\sum_{k'} C^j_{kk'} I^{k'}_{jj} + \sum_i \sum_{k'} C^j_{kk'} I^{k'}_{ij} \right] \tag{11}$$

$$\lambda^k_{ji} = \frac{\beta}{N^*_i} \left[\sum_{k'} C^i_{kk'} I^{k'}_{ii} + \sum_l \sum_{k'} C^i_{kk'} I^{k'}_{li} \right] \tag{12}$$

Where β is the probability of becoming infected as a result of a single contact, and N^*_j is the effective number of individuals in comuna j (said differently, the actual number of individuals staying in comuna j). This is, by definition, the sum of the number of individuals from j actually located in j and the number of individuals from a connected comuna i located in j, namely $N_j = N_{jj} + \sum_{i \neq j} N_{ij}$. In the time scale separation, this expression can be written as:

$$N^*_j = \frac{N_j}{1 + \sigma_j/\tau} + \sum_i \frac{N_i \sigma_{ij}/\tau}{1 + \sigma_i/\tau}. \tag{13}$$

Substituting the equilibrium values resulting from the time scale separation also for $I^{k'}_{jj}$ and $I^{k'}_{ij}$ we obtain:

$$\lambda^k_{jj} = \frac{\beta}{N^*_j} \left[\sum_{k'} C^j_{kk'} \frac{I^{k'}_j}{1 + \sigma_j/\tau} + \sum_i \sum_{k'} C^j_{kk'} \frac{I^{k'}_i \sigma_{ij}/\tau}{1 + \sigma_i/\tau} \right] \tag{14}$$

$$\lambda^k_{ji} = \frac{\beta}{N^*_i} \left[\sum_{k'} C^i_{kk'} \frac{I^{k'}_i}{1 + \sigma_i/\tau} + \sum_l \sum_{k'} C^i_{kk'} \frac{I^{k'}_l \sigma_{li}/\tau}{1 + \sigma_l/\tau} \right] \tag{15}$$

Then, the total force of infection λ^k_j can be written as the the sum of these two contributions weighted, respectively, by the probability of finding a susceptible in age group k from comuna j in the home

Supplementary Table 3: **Transitions between compartments**

Transition	Type	Rate
$S_j^k \to L_j^k$	Contagion	λ_j^k
$L_j^k \to I_j^k$	Spontaneous	ϵ
$I_j^k \to R_j^k$	Spontaneous	μ

population ($S_{jj}^k/S_j^k = 1/(1+\sigma_j/\tau)$ using Eq. 9), and in a connected comuna i ($S_{ji}^k/S_j^k = (\sigma_{ji}/\tau)/(1+\sigma_j/\tau)$ using Eq. 10):

$$\lambda_j^k = \frac{\lambda_{jj}^k}{1+\sigma_j/\tau} + \sum_i \frac{\lambda_{ji}^k \sigma_{ji}/\tau}{1+\sigma_j/\tau} \tag{16}$$

The impact of restrictions. Mobility restrictions are implemented changing the mobility matrix $\boldsymbol{\sigma}$ at the start of the partial and the full lockdown. Contacts reduction, instead, is implemented multiplying the contacts matrix C^j of each comuna by the respective parameter. For example, the general element of the contacts matrix C^j during the partial lockdown for comuna j is $r_j^{partial} C_{kk'}^j$.

5 Mobile Phone Users Geographical Distribution

Supplementary Figure 13 shows a scatter plot of the number of mobile users in our dataset and the resident population from official census (2017) for the 342 comunas of continental Chile. Because of the agreements with mobile phone data provider, Telefónica Movistar, we cannot report raw numbers, therefore we have normalized values with respect to their maximum. We observe a high Pearson correlation of $\rho = 0.97$, indicating that the geographical distribution of our user base well represents the real one.

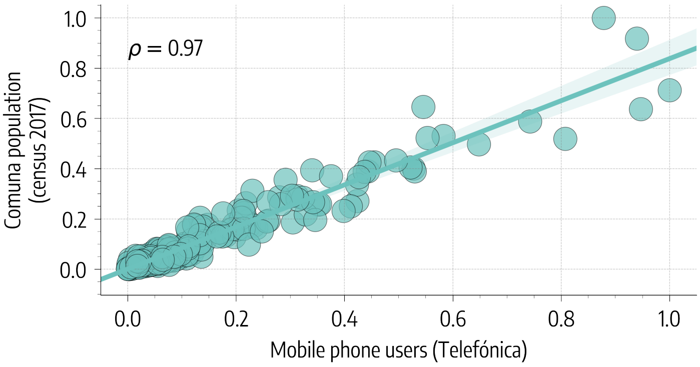

Supplementary Figure 13: **Correlation between Telefónica Movistar mobile phone users and official census.** We show the correlation in the 342 comunas in continental Chile. We display the regression line, 95% CI and the Pearson correlation coefficient ρ.

References

[1] Departamento de Estadisticas e Información de Salud. `https://deis.minsal.cl/#datosabiertos` (2020).

[2] Klugman, J. Human development report 2010 – 20th anniversary edition. the real wealth of nations: Pathways to human development (2010).

[3] Chinazzi, M. *et al.* The effect of travel restrictions on the spread of the 2019 novel coronavirus (COVID-19) outbreak. *Science* **368**, 395–400 (2020).

[4] Lai, S. *et al.* Effect of non-pharmaceutical interventions to contain COVID-19 in China. *Nature* (2020).

[5] Wu, J., Leung, K. & Leung, G. Nowcasting and forecasting the potential domestic and international spread of the 2019-nCoV outbreak originating in wuhan, china: a modelling study. *The Lancet* **395** (2020).

[6] Di Domenico, L., Pullano, G., Sabbatini, C. E., Boëlle, P.-Y. & Colizza, V. Impact of lockdown on COVID-19 epidemic in Île-de-France and possible exit strategies. *BMC Medicine* **18**, 240 (2020).

[7] Hao, X. *et al.* Reconstruction of the full transmission dynamics of covid-19 in wuhan. *Nature* **584** (2020).

[8] Lauer, S. A. *et al.* The incubation period of coronavirus disease 2019 (covid-19) from publicly reported confirmed cases: Estimation and application. *Annals of Internal Medicine* **172**, 577–582 (2020). PMID: 32150748.

[9] Ferretti, L. *et al.* Quantifying sars-cov-2 transmission suggests epidemic control with digital contact tracing. *Science* **368** (2020).

[10] Mizumoto, K., Kagaya, K., Zarebski, A. & Chowell, G. Estimating the asymptomatic proportion of coronavirus disease 2019 (covid-19) cases on board the diamond princess cruise ship, yokohama, japan, 2020. *Eurosurveillance* **25** (2020).

[11] Lavezzo, E. *et al.* Suppression of a sars-cov-2 outbreak in the italian municipality of vo'. *Nature* **584** (2020).

[12] Li, R. *et al.* Substantial undocumented infection facilitates the rapid dissemination of novel coronavirus (sars-cov-2). *Science* **368**, 489–493 (2020).

[13] Backer, J. A., Klinkenberg, D. & Wallinga, J. Incubation period of 2019 novel coronavirus (2019-nCoV) infections among travellers from wuhan, china, 20–28 january 2020. *Eurosurveillance* **25** (2020).

[14] Kissler, S. M., Tedijanto, C., Goldstein, E., Grad, Y. H. & Lipsitch, M. Projecting the transmission dynamics of SARS-CoV-2 through the postpandemic period. *Science* **368**, 860–868 (2020).

[15] Goscé, L., Phillips, P. A., Spinola, P., Gupta, D. R. K. & Abubakar, P. I. Modelling sars-cov2 spread in london: Approaches to lift the lockdown. *Journal of Infection* **81**, 260 – 265 (2020).

[16] Walker, P. G. *et al.* The impact of covid-19 and strategies for mitigation and suppression in low-and middle-income countries. *Science* **369**, 413–422 (2020).

[17] Google LLC "Google COVID-19 Community Mobility Reports". `https://www.google.com/covid19/mobility/` (2020).

[18] Hale, T. *et al.* A global panel database of pandemic policies (oxford covid-19 government response tracker). *Nature Human Behaviour* 1–10 (2021).

[19] Oxford covid-19 government response tracker. `https://www.bsg.ox.ac.uk/research/research-projects/coronavirus-government-response-tracker#data` (2020).

[20] Balcan, D. *et al.* Seasonal transmission potential and activity peaks of the new influenza A(H1N1): A Monte Carlo likelihood analysis based on human mobility. *BMC medicine* **7**, 45 (2009).

[21] Keeling, M. J. & Rohani, P. Estimating spatial coupling in epidemiological systems: a mechanistic approach. *Ecology Letters* **5**, 20–29 (2002).

[22] Sattenspiel, L. & Dietz, K. A structured epidemic model incorporating geographic mobility among regions. *Mathematical Biosciences* **128**, 71–91 (1995).
