# Supplementary material for: Estimating the effect of social inequalities on the mitigation of COVID-19 across communities in Santiago de Chile
Source: Nat Commun. 2021 Apr 23;12:2429. doi: 10.1038/s41467-021-22601-6 (PMC8065143; doi:10.1038/s41467-021-22601-6)
Supplement: Supplementary file 3 — Reporting Summary [file 41467_2021_22601_MOESM3_ESM.pdf]

nature research

Corresponding author(s): Nicola Perra
Last updated by author(s): 2021/03/12

# Reporting Summary

Nature Research wishes to improve the reproducibility of the work that we publish. This form provides structure for consistency and transparency in reporting. For further information on Nature Research policies, see our Editorial Policies and the Editorial Policy Checklist.

## Statistics

For all statistical analyses, confirm that the following items are present in the figure legend, table legend, main text, or Methods section.

| n/a | Confirmed | |
|---|---|---|
| ☐ | ☒ | The exact sample size ($n$) for each experimental group/condition, given as a discrete number and unit of measurement |
| ☐ | ☒ | A statement on whether measurements were taken from distinct samples or whether the same sample was measured repeatedly |
| ☒ | ☐ | The statistical test(s) used AND whether they are one- or two-sided<br>*Only common tests should be described solely by name; describe more complex techniques in the Methods section.* |
| ☐ | ☒ | A description of all covariates tested |
| ☐ | ☒ | A description of any assumptions or corrections, such as tests of normality and adjustment for multiple comparisons |
| ☐ | ☒ | A full description of the statistical parameters including central tendency (e.g. means) or other basic estimates (e.g. regression coefficient) AND variation (e.g. standard deviation) or associated estimates of uncertainty (e.g. confidence intervals) |
| ☒ | ☐ | For null hypothesis testing, the test statistic (e.g. $F$, $t$, $r$) with confidence intervals, effect sizes, degrees of freedom and $P$ value noted<br>*Give P values as exact values whenever suitable.* |
| ☐ | ☒ | For Bayesian analysis, information on the choice of priors and Markov chain Monte Carlo settings |
| ☒ | ☐ | For hierarchical and complex designs, identification of the appropriate level for tests and full reporting of outcomes |
| ☐ | ☒ | Estimates of effect sizes (e.g. Cohen's $d$, Pearson's $r$), indicating how they were calculated |

*Our web collection on statistics for biologists contains articles on many of the points above.*

## Software and code

Policy information about availability of computer code

| | |
|---|---|
| Data collection | The data was collected by Telefonica by considering tower registering activity. |
| Data analysis | The mobility and contact data was generated using codes written in BigQuery SQL on Google Cloud. The main epidemic model described in the manuscript has been implemented with the programming language C++. The alternative models, detailed in Section 2 of the Supplementary Information, have been implemented in C++ or in python. All other analysis of the model's output are done in python.<br>In this Github https://github.com/ngozzi/covid19-santiago as well as in this Zenodo link https://zenodo.org/record/4605505 we uploaded the codes to run the epidemic models as well as the relative information |

For manuscripts utilizing custom algorithms or software that are central to the research but not yet described in published literature, software must be made available to editors and reviewers. We strongly encourage code deposition in a community repository (e.g. GitHub). See the Nature Research guidelines for submitting code & software for further information.

## Data

Policy information about availability of data

All manuscripts must include a data availability statement. This statement should provide the following information, where applicable:

- Accession codes, unique identifiers, or web links for publicly available datasets
- A list of figures that have associated raw data
- A description of any restrictions on data availability

The raw data analysed in the study are not publicly available due to privacy reasons. All the aggregated mobility data needed to run the model are available at https://github.com/ngozzi/covid19-santiago. Analysis of the anonymised mobile phone data was performed on mobile operator’s systems without transferring it outside. Only aggregated mobility patterns across municipalities were provided to researchers outside Chile and only these have been used for the results presented here.

# Field-specific reporting

Please select the one below that is the best fit for your research. If you are not sure, read the appropriate sections before making your selection.

☐ Life sciences ☒ Behavioural & social sciences ☐ Ecological, evolutionary & environmental sciences

For a reference copy of the document with all sections, see nature.com/documents/nr-reporting-summary-flat.pdf

# Behavioural & social sciences study design

All studies must disclose on these points even when the disclosure is negative.

| | |
|---|---|
| Study description | We adopted quantitative methods to extract mobility as well as contacts reduction information between and within comunas (municipalities) from mobile phone data in Santiago de Chile. We used this data to estimate the variation of mobility patterns across and contacts within comunas (i.e., municipalities). Furthermore, the data is used in a metapopulation and age-structured epidemic model to characterize the unfolding of SARS-CoV-2 in the region, understand the effects and impacts of non-pharmaceutical interventions put in place. We also developed and tested simpler epidemic models as a way to highlight the importance of including detailed data about mobility and contacts. |
| Research sample | The sample includes all Telefonica Movistar users in the Santiago de Chile metropolitan area: 1.4 M users. This constitutes 22% of the total population in the area. While no socio-demographic information has been made available to us nor used in the article, the Pearson correlation coefficient of census data and "home location", as described in the main manuscript, is 0.96. We added this information in the discussion and a detailed plot in the SI.<br>Furthermore, the Telefonica Movistar data has been found to well represent the different socio-demographic groups of Santiago in previous publications such as:<br>Laetitia Gauvin, et al. Gender gaps in urban mobility. Humanities and Social Sciences Communications, 7(1):1, 13, 2020.<br>More details are provided in the article |
| Sampling strategy | We considered all users with a home antenna within Santiago de Chile: 1.4 M users. We assign a home antenna to each device by finding the most active antenna during night hours. Individuals' mobility traces have been aggregated to quantify the mobility among comunas, and contacts reduction between individuals. Mobility describes the (varying) rates at which people travel among different comunas, while contacts reduction parameters estimate to what extent physical contacts varied in time in each comuna. It is important to stress how only aggregated mobility patterns across municipalities were provided to researchers outside Chile and only these have been used for the results presented here. |
| Data collection | We use phone data in the form of eXtended Detail Records (XDR). This stream records every interaction (e.g., packet request) between devices and antennas. The data was collected, recorded and stored by Telefonica Movistar for their business operations. An entry in our dataset can be formalized as a tuple (d,t,k) indicating a packet request to antenna k made by device d at time t. We approximate the position of d with that of the antenna, which has a fixed latitude and longitude. The data has been used to generate the aggregated mobility patterns across comunas which are those used in the article |
| Timing | We considered data collected between 2020/02/27 - 2020/06/01 |
| Data exclusions | No data or user was excluded |
| Non-participation | All users where considered |
| Randomization | Due to the strict regulations, privacy and ethical concerns only aggregated data was made available by Telefonica Movistar and used in this research. Hence, the data available does not allow randomization of individual mobility traces. |

# Reporting for specific materials, systems and methods

We require information from authors about some types of materials, experimental systems and methods used in many studies. Here, indicate whether each material, system or method listed is relevant to your study. If you are not sure if a list item applies to your research, read the appropriate section before selecting a response.

## Materials & experimental systems

| n/a | Involved in the study | |
|---|---|---|
| ☒ | ☐ | Antibodies |
| ☒ | ☐ | Eukaryotic cell lines |
| ☒ | ☐ | Palaeontology and archaeology |
| ☒ | ☐ | Animals and other organisms |
| ☒ | ☐ | Human research participants |
| ☒ | ☐ | Clinical data |
| ☒ | ☐ | Dual use research of concern |

## Methods

| n/a | Involved in the study | |
|---|---|---|
| ☒ | ☐ | ChIP-seq |
| ☒ | ☐ | Flow cytometry |
| ☒ | ☐ | MRI-based neuroimaging |
